# De‐Coupled Water and Nitrogen Translocation From Subsoil to Canopy of Temperate Forest Trees

**DOI:** 10.1111/pce.70327

**Published:** 2025-12-15

**Authors:** Klara Mrak, Christina A. Hackmann, Sharath S. Paligi, Christian Ammer, Norbert Lamersdorf, Christoph Leuschner, John D. Marshall, Ruth‐Kristina Magh

**Affiliations:** ^1^ Soil Science of Temperate Ecosystems University of Göttingen Göttingen Germany; ^2^ Silviculture and Forest Ecology of the Temperate Zones University of Göttingen Göttingen Germany; ^3^ Plant Ecology, Albrecht von Haller Institute for Plant Sciences University of Göttingen Göttingen Germany; ^4^ Centre for Biodiversity and Sustainable Land Use (CBL) University of Göttingen Göttingen Germany; ^5^ Department of Earth Sciences University of Gothenburg Gothenburg Sweden; ^6^ Terrestrial Ecohydrology, Institute of Geoscience Friedrich Schiller University Jena Jena Germany

**Keywords:** dual‐isotope tracer, nutrient uptake, sap flow, temperate forest

## Abstract

Water and nitrogen (N) transport from soil to canopy play a central role in tree functioning, yet direct evidence for their timing and coupling in mature forests remains scarce. We report results from a paired dual‐isotope (^2^H, ^15^N) tracer experiment in a temperate forest, comparing water and nitrate uptake patterns across tree species (Douglas fir, European beech), soil textures (loamy, sandy) and rooting depths (surface, subsoil). Using simultaneous double tracer injections into the lower rooting zone, followed by canopy xylem sap and foliage sampling over 2 months, we quantified the appearance of water and nitrate in xylem sap and foliage, supported by sap flow measurements to estimate transit times. Water reached the canopy faster than nitrogen, revealing a marked asynchrony in resource translocation for both species and sites—the first such field observation. Douglas fir showed greater subsoil water and nitrate uptake on sandy than on loamy soil, and higher subsoil uptake compared to beech under identical conditions. Tree species, soil texture and soil depth jointly govern water and nitrate uptake dynamics, as well as their temporal decoupling. These findings provide novel field‐based evidence of asynchronous water and nitrate transport, advancing our understanding of tree ecophysiology and nutrient cycling under field conditions.

## Summary statement

Dual‐isotope tracing in situ shows faster transport of water than nitrate from soil to canopy of mature trees, indicating effects of root function, soil texture and species traits. Douglas fir exhibits greater subsoil water and nitrate uptake on sandy than on loamy soils and higher uptake than beech under similar soil conditions.

## 1 Introduction

The relationship between water and nitrogen (N) uptake in trees is a result of complex interactions and species‐specific responses. In recent decades, tree response to drought on the one hand, and different levels of nutrient supply on the other hand, have been a focus of ecophysiological research (Gessler et al. 2017; Grossiord et al. 2018; Schönbeck et al. 2021; Paligi et al. 2024). Studies have shown that tree N nutrition depends on soil water availability (Simon et al. 2017) and that the sensitivity of trees to soil desiccation is at least partially due to impaired N acquisition (Geßler et al. 2005; Dannenmann et al. 2016). Only a few empirical studies have directly addressed the interaction of the water and the nitrogen cycle in the tree‐soil sphere. If so, conclusions were drawn for water and nitrogen for separate tree compartments only (Kulmatiski et al. 2017; Henriksson et al. 2021; Lutter et al. 2021). None thus far has studied water and nutrient translocation from soil to canopy of mature trees in situ in real‐time; therefore, a knowledge gap exists concerning the coupling of these processes and to what extent it depends on tree species and soil type.

The popularity of non‐native Douglas fir (DF) (*Pseudotsuga menziesii* [Mirbel] Franco) in Central Europe is growing due to its fast growth, high wood quality and favourable ecological properties (Lévesque et al. 2014; Vitali et al. 2017; Thomas et al. 2022). It is discussed as a possible alternative to drought‐sensitive Norway spruce (*Picea abies* [L.] H. Karst.) (Fuchs et al. 2022). European beech (*Fagus sylvatica* L., hereafter beech, B), which is the dominant broadleaved tree species in natural forests of Central Europe (Giesecke et al. 2007), has a slower growth rate and a lower economic return (Martinez del Castillo et al. 2022), but exhibits higher drought resistance than Norway spruce and an efficient belowground resource acquisition (Zang et al. 2014; Leuschner 2020; Hackmann et al. 2024; Paligi et al. 2024). Norway spruce is known as a shallow‐rooted species with most fine roots in the organic layer and the upper mineral soil (Köstler et al. 1968; Lwila et al. 2021). European beech and DF develop heart‐shaped root systems that reach 1–2 m and deeper (Köstler et al. 1968; Hendriks and Bianchi 1995; Spielvogel et al. 2014), nevertheless most of their roots are concentrated in the upper 30–50 cm of mineral soil (Meier et al. 2018; Lwila et al. 2021; Pietig et al. 2026). Deeper roots allow access to resources from deeper soil layers, a crucial trait under drought (Kahmen et al. 2022), that influences both water and nutrient cycling in forest ecosystems (Gessler et al. 2017). While both beech and DF can use resources from deeper soil depths than spruce (Nadezhdina et al. 2014; Goisser et al. 2016; Hackmann et al. 2025), the differences between their resource acquisition strategies have barely been investigated. In terms of water uptake depth, the two species showed no significant difference across different sites (Hackmann et al. 2025). Furthermore, previous research found high nitrate concentrations in soil solution beneath the main rooting zone (60 cm soil depth) and linked it to species identity that is, DF on loamy soils, while no leaching was found under beech (Mrak et al. 2024).

Tree species identity effects are strongly modulated by site conditions (Ammer 2019; Mrak et al. 2024), which can result in large trait heterogeneity of a species across sites (Brinkmann et al. 2018; Castro‐Díez et al. 2021). Local climate and soil conditions influence root traits (Lwila et al. 2021; Wankmüller et al. 2024), microbial (Lu and Scheu 2021) and mycorrhizal communities (Likulunga et al. 2021) and the relative importance of mass flow and diffusion for nutrient transport (Oyewole et al. 2014). These interactions shape tree water and nutrient acquisition and therefore the tree's resource use efficiency (Ahrens et al. 2020; Agee et al. 2021). In sandy and/or nutrient‐poor soils, trees tend to develop more expansive root systems (Schenk and Jackson 2002; Kleinert et al. 2018). While this suggests a higher potential for deeper water and nutrient uptake on sandy than on loamy soils, such site effects have not yet been studied for DF. In mixed‐species forests, uptake traits of functionally dissimilar species may lead to complementary resource use, which can be more pronounced on sandy, resource‐limited sites (Forrester et al. 2017; Mina et al. 2018; Ammer 2019).

Root water uptake is mainly driven by transpiration, which creates mass flow of water between soil and root (Steudle 2000; McMurtrie and Näsholm 2018). Water can traverse cell membranes both actively (e.g., mediated by aquaporines) and passively. This is not the case for nutrient ions (such as nitrate) (Steudle and Peterson 1998). While the flux of N to the root surface is governed by mass flow and diffusion (Oyewole et al. 2014), the uptake of N into roots is an active process. It requires energy, and involves transporter presence and activity to overcome the positive concentration gradient of a nitrogenous compound from outside the root to the inside (Pfautsch et al. 2009). The root tip, composed of plant and mycorrhizal tissue, retains more ammonium than nitrate, meaning that a higher proportion of nitrate compared to ammonium enters the plant tissue (Leberecht et al. 2016). In the root, nitrate is either metabolised into reduced forms, that is, ammonium or amino acids (Finlay et al. 1989; Geβler et al. 2004; Yoneyama and Suzuki 2019), or directly loaded into the xylem and transported to photosynthetically active and storage organs, while some nitrogenous compounds are also circulated back into the roots, as a signal to up‐ or down‐regulate uptake transporters (Rennenberg et al. 1998). These processes are widely recognised at a theoretical and molecular level (Oyewole et al. 2014; McMurtrie and Näsholm 2018; Tegeder and Masclaux‐Daubresse 2018), yet the consequences at mature tree level under field conditions remain to be shown.

Although root biomass is prerequisite for water and nutrient uptake, roots can be present and still not function (Weemstra et al. 2016; Kulmatiski et al. 2017). Isotopic tracers are an effective tool to assess the active part of the root system. Water stable isotopes (^2^H, ^18^O) are frequently used to infer water sources and root water uptake depth, applying both labelling and natural abundance approaches (Ehleringer and Dawson 1992; Dubbert and Werner 2019). The natural abundance of ^15^N provides insights into plant‐internal N cycling and uptake processes (Cernusak et al. 2009; Peuke et al. 2013; Cui et al. 2020), but not N uptake depth. To address this, studies have used ^15^N labelling to link N uptake to its source depth (Rowe et al. 1998; Göransson et al. 2008; Kulmatiski et al. 2017; Kulmatiski et al. 2020; Byers et al. 2024). These studies investigated N fixed in sink tissue (foliage), and not in the transient N pool in xylem sap. Therefore, tracer studies investigating the origin depth of this transient water and N pool, reflecting the uptake process from root to canopy in mature trees, are lacking. Field‐based data on uptake patterns yields valuable information about species‐specific strategies and site effects on water and N uptake, as well as the temporal coupling of water and N transport.

Sampling tree xylem water for both ^15^N and ^2^H presents a challenge, as standard methods for water isotopes like cryogenic vacuum distillation (Koeniger et al. 2011), are unsuitable for nitrogen analysis. Previous studies have analysed foliage (for ^15^N) and the outermost tree ring (for ^2^H) after the growing season to track a pulse of ^2^H and ^15^N tracers (Henriksson et al. 2021; Lutter et al. 2021), however, this yields neither information about the temporal aspect of the uptake nor does it analyse the same compartment of the tree for both isotopes. The Scholander pressure chamber (Scholander et al. 1965), commonly used for measuring plant water potential, has been employed to extract xylem sap for water (Geißler et al. 2019; Magh et al. 2020) and N isotope analysis (Hu and Guy 2020). We adapted this method to simultaneously analyse both isotopes in twig sap of mature forest trees.

Previous isotopic labelling studies have shown that the travel time of the ^2^H signal from soil via xylem to the canopy of mature temperate forest trees is at the scale of weeks (Meinzer et al. 2006; Magh et al. 2020; Tetzlaff et al. 2021; Hackmann et al. 2025). This travel time can be approximated by measuring sap velocity (Seeger and Weiler 2021; Brighenti et al. 2024), an approach that we used to schedule our sampling campaign after tracer application.

In this study, we applied a combined ^2^H and ^15^N tracer pulse at 60 cm soil depth to mature DF and European beech trees in temperate forest stands, differing in soil conditions (i.e., sandy vs. loamy). We estimated travel times to the canopy with sap flow measurements, and accordingly sampled twig xylem water 4, and 6 weeks after labelling. In addition, we sampled current‐year foliage at the same time points as xylem water and once after the vegetative season.

With the dual‐labelling approach, we aimed to directly assess the magnitude and temporal coupling of water and nutrient uptake by mature trees from below the main rooting zone (subsoil), (1) for DF on two contrasting sites (sandy and loamy), and (2) comparing DF and European beech under the same site conditions (sandy). Based on the current state of research on root distribution, nitrate accumulation in soil solution, and water and N transport in plant and soil for the studied sites and species, we hypothesised that:

1. After the labelling pulse, the ^15^N tracer will be found later than the ^2^H tracer in xylem sap due to the decoupling,
2. Site determines subsoil nitrate and water uptake of DF, with higher uptake on the sandy than on the loamy site,
3. Beech and DF take up similar amounts of subsoil water and nitrate.

## 2 Materials and Methods

### 2.1 Study Sites

Research was conducted at two temperate forest sites in north‐western Germany: Winnefeld (ca. 20% sand in the mineral topsoil, ‘loamy’), and Unterlüß (ca. 80% sand in the mineral topsoil, ‘sandy’; lower precipitation, higher temperatures). Detailed site characteristics can be found in Table 1.

**Table 1:** Location and characteristics of the study sites. Long‐term climatic averages (MAP, mean annual precipitation and MAT, mean annual temperature) are based on elevation‐sensitive interpolations from weather stations of the German Weather Service (DWD) 1990–2017. Soil texture (10–30 cm mineral soil depth) was assessed by Foltran et al. (2023). N deposition by bulk precipitation shows mean of years 2012–2021 of close‐by monitoring plots (ICP Forests Intensive Monitoring, Level II; sandy Luess; site 301 and loamy Solling; site 304, 305) by the Northwest German Forest Research Institute (Meesenburg, pers. comm.), while *N* deposition via throughfall was measured by Mrak et al. (2024) (mean of year 2020–2022).

Site	Location (Lat., Long.)	Elevation (m.a.s.l.)	MAP (mm)	MAT (°C)	Soil type (topsoil texture)	N deposition by bulk precipitation (kg ha^−1^ a^−1^)	N deposition by throughfall (kg ha^−^^1^ a^−1^)
Winnefeld (‘loamy’)	51.664759, 9.571193	345–402	839	8.8	Dystric cambisol (20% sand, 57% silt, 23% clay)	10.03	16.95
Unterlüß (‘sandy’)	52.836752, 10.330936	149–167	747	9.0	Haplic podzol (79% sand, 15% silt, 6% clay)	8.74	15.40

At both locations, study plots of 50 × 50 m were established in mature, even‐aged forest stands (average age 80–90 years, tree heights ca. 25–35 m) with a closed canopy. The stands are regularly managed and thinned according to common continuous cover forestry practice in Germany; no thinning occurred in the last 10–15 years, resulting in an average basal area of 33.0 m^2^ ha^−1^ in loamy Winnefeld and of 35.6 m^2^ ha^−1^ in sandy Unterlüß.

We investigated two tree species: Douglas fir (DF) and European beech (B). Trees were measured on four plots: three plots at the sandy site (one pure stand of B, one pure stand of DF, and a mixed stand of the two), and one pure DF plot at the loamy site.

While the sandy site lies in the lowlands (groundwater depth > 30 m), the loamy site is part of the Weser uplands, with the plot located on the shoulder of a hill. Thus, we assume neither groundwater access nor considerable lateral flow and therewith no lateral input of N into the rooting zone. Based on 5 years of continuous measurements of soil volumetric water content up to 100 cm soil depth, there are no signs of stagnant soil water, so that an accumulation of nitrate in the subsoil can be excluded. A fine root inventory showed that DF (similar to beech) exhibited higher fine root biomass, specific root length and root area index at the sandy compared to the loamy site, even though the differences were no longer significant at deeper soil layers (Lwila et al. 2021, Figure S1).

### 2.2 Plant and Environmental Conditions

#### 2.2.1 Midday Xylem Water Potential (Ψ_MD_)

To assess tree water status, we measured midday twig xylem water potential (Ψ_MD_) each time xylem water was sampled (0, 4, 5 and 6 weeks after labelling) on twigs of the same branch. A terminal twig with 3–5 leaves (in case of beech), respectively with current‐year needles (in case of DF) was used to measure Ψ_MD_ within 5 min from cutting the branch, right before the xylem water was extracted, between 10 AM and 2 PM.

#### 2.2.2 Sap Flow

We estimated sap velocity up to sampling height (25 m for DF, 20 m for beech) from measurements of heat pulse velocity sap flow sensors (HPV06, Implexx Sense, Melbourne, Australia) following the protocol detailed in Paligi et al. (2025). Briefly, sensors were installed at breast height (1.3 m above ground) on the 12 sample trees at the sandy site (for the loamy site no sap flow data was available). For the installation, we removed at least 0.5 cm bark to ensure the measurement points were at 0.5 and 1.5 cm depth in the sap wood. Data were logged every 30 min in a CR1000 datalogger (Campbell Scientific, USA). Time dependent probe misalignment was corrected following the saturation method (Forster 2019; Paligi et al. 2025) using IMPLEXX‐SF Software v.1 (Implexx Sense, Melbourne, Victoria, Australia).

Since measurements in the outer sap wood may not represent the overall sap velocity and thus lead to overestimation of travel times, we used the radial sap flow dataset obtained from the heat field deformation sensors (HFD100, ICT International Pty Ltd., Armidale, Australia) on the same trees to determine tree‐specific relative radial sap flux density profiles (Figure S2). Based on these, we estimated maximum radial sap flux densities for each time point, which were summed up to obtain daily sap velocity (m). The cumulative sap velocities after the day of labelling were used as an indicator for the potential tracer travel height within the tree, with the aim to capture the time point of tracer arrival when sampling xylem water in the canopy.

#### 2.2.3 Environmental Conditions

Volumetric soil water content (SWC, %) and soil matric potential (SMP, MPa) were measured every hour using time‐domain reflectometry probes (Campbell Scientific, Logan, Utah, USA) and dielectric water potential sensors (Teros 21, Meter Environment, Washington, USA), respectively, installed in all plots at depths 5, 20, 50 and 100 cm 4 years prior to the experiment. SMP was corrected for temperature according to (Walthert and Schleppi 2018). Air temperature (°C) and open sky precipitation (mm) were measured and stored at 15‐min resolution intervals (Campbell CR300) at two climate stations (MET300; DMS, Dundee, UK) close to the respective sites. Long‐term averages (1991–2017) of precipitation and air temperature per site were based on elevation‐sensitive interpolations of weather stations of the German Weather Service (DWD) (see Ammer et al. [2020] for details).

#### 2.2.4 Ammonium and Nitrate in the Soil and Soil Solution

To estimate the plant‐available fraction of ammonium and nitrate, soil was sampled at 10 cm mineral soil depth in three subsamples per plot in June 2022. We extracted 20 g of fresh soil in 40 mL 1 mM CaCl_2_ solution, shook it horizontally for 30 min, filtered it through a folded filter paper MN 280 (Macherey‐Nagel, Düren, Germany), further through a 0.2 µm syringe filter and stored at −20°C until analysis. The simultaneous analysis for nitrate‐N, ammonium‐N and total N was performed on a SEAL continuous flow analyser AA3 HR (SEAL Analytical GmbH, Norderstedt, Germany). The nitrate and ammonium concentrations in soil solution were measured by continuous collection of soil solution between 1 June 2021 and 10 July 2021 using P‐80 suction lysimeters (Bredemeier et al. 1990), installed at 5 cm soil depth with three subsamples on the plot, as described in Mrak et al. (2024), and analysed using the same methodology as for the soil extracts (SEAL continuous flow analyser).

### 2.3 Tracer Application and Isotopic Sampling

#### 2.3.1 Tracer Application

We labelled soil at 60 cm mineral soil depth around 15 trees (hereafter ‘subsoil labelling’, *n* = 3 per species per plot). In addition, we applied the same tracer on the mineral soil surface, below the litter layer, around three DF trees nearby the DF plot at the loamy site (hereafter ‘surface labelling’). This treatment did not address the main research questions, but served as a proof of concept. Since in the topsoil, unlike in the subsoil, it was certain that active fine roots were present (Lwila et al. 2021), we aimed to verify that the tracer at the given amount and concentration was generally detectable in the tree. The loamy site was chosen for this test, as fine‐textured soils have lower hydraulic conductivity and slower percolation rates, yielding a more conservative and robust validation of the method. A conceptual figure of the study design is depicted in Figure S3.

For the subsoil labelling, we installed eight hard plastic tubes (Ø 20 mm) in a circle with 2 m distance from the stem and applied the tracer solution by slowly pouring it into a funnel inserted into the tube, so that the only pressure exerted on the solution was gravity (the process took 20 min per tube). The tubes were installed ca. 2 months before the experiment to allow roots and soil to recover from the disturbance; we first created a hole of 60 cm depth with a Pürckhauer soil corer (Ø 22 mm, MMM tech support GmbH, Berlin), and then gently manually inserted the tubes. On the mixed plot, some tube positions between trees were overlapping, resulting in a total number of 132 tubes (Figure S3).

The tracer was applied in deionized water containing both tracers (^2^H and ^15^N). The solution was prepared in 144 new plastic bottles of 330 mL volume, each containing 314 mL of the tracer solution. Each tree received eight bottles of tracer solution; one bottle per tube on the pure plots, and slightly more per tube on the mixed plot, equally distributed across the overlapping tubes. This corresponded to 169.88 mg ^15^N (1.2 g K^15^NO_3_ at 99.9 atom‐% ^15^N) and 2407 mL water with a deuterium concentration of 1.959 atom‐% (127 300 δ‰ V‐SMOW). To achieve this, we combined 5 mL of pre‐prepared solution containing the ^15^N tracer with 5.84 mL of 99.9% deuterated water and 290 mL deionized water with known deuterium concentration (0.0164 atom‐% ^2^H; −55.74 δ‰ ^2^H V‐SMOW) in each bottle. The final concentration of N in the tracer solution was 67.695 mg/L.

#### 2.3.2 Xylem Water Sampling

Based on tracer travel time estimations from sap flow data, we sampled xylem water 4–6 weeks after tracer application: on the 7, 14 and 21 August (sandy site), and on the 8, 15 and 22 August (loamy site).

At each sampling campaign, three to four sun‐exposed, vital branches (30–70 cm long) were accessed by tree climbing, cut and delivered undamaged to the extraction point. Xylem water was extracted within 1 h from sampling and was stored in opaque bags to prevent desiccation during subsequent sampling. Smaller twigs (ca. 0.5 cm diameter) were cut from the sample, and bark, phloem and cambium were removed from the last 0.5 cm of the cut part with a clean and dry blade, to ensure only xylem sap was sampled. The twig was then inserted into a Scholander pressure chamber (PMS Instruments, Corvallis, Oregon, USA), and a silicone tube (new for each sample) was attached onto the peeled part. A maximum pressure of up to 3 MPa was applied to extract xylem water, which was 0.5–1 MPa higher than the measured twig water potential. The xylem sap was then collected through the silicone tube into a 2 mL Eppendorf tube. Multiple pressure chambers were deployed at the same time to process samples simultaneously. Samples were immediately stored at 4°C until further processing. The extracted sample was split into two subsamples that were used for ^2^H and ^15^N analysis, respectively.

#### 2.3.3 Foliage Sampling

Current‐year foliage (ca. 20 g dry weight) was collected at the same time points as xylem water, and one additional time towards the end of the growing season, after 14 weeks (the 16 and 17 October). The samples were dried at 60°C for 72 h, milled to fine powder using a ball mill (Retsch, Düsseldorf, Germany) and weighed into tin capsules. For phosphorus (P) content, milled samples were analysed by ICP‐OES after a pressure digestion with 65% HNO_3_.

#### 2.3.4 Throughfall Sampling

To determine the local meteoric water line (LMWL), we sampled throughfall with custom‐built rain collectors as described in Prechsl et al. (2014). On five nearby plots per site, one collector was installed in spring 2021 and sampled after any substantial rain event (summer 2021), and monthly afterwards (until summer 2023). The data were combined to calculate the LMWL, based on 157 and 93 datapoints for the sandy and the loamy site, respectively.

#### 2.3.5 Isotope Analysis and Calculations

For ^15^N, a total of 1 mL sample was applied to a tin capsule and allowed to evaporate at 60°C (in five droplets of 0.2 mL each), leaving the residue in the tin capsule. These capsules, as well as the capsules containing foliage samples, were then analysed for N isotopic composition on a Delta Plus mass spectrometer (Finnigan MAT, Bremen, Germany; Interface: Conflo III, Finnigan MAT), coupled with an elemental analyser (NA2500, CE Instruments, Rodano, Milan, Italy). For ^2^H and ^18^O in xylem and throughfall water, samples were measured on a high temperature elemental analyser (TC/EA, Thermo Electron Corporation, Bremen, Germany) coupled via a Con‐Flo III interface to a Delta V Plus isotope ratio mass spectrometer (Thermo Electron Corporation, Bremen, Germany). Both measurements were done at the Center for Stable Isotope Research and Analysis (KOSI, Georg‐August‐University Göttingen, Germany).

Isotope ratios were expressed using the δ notation:

$$\delta_{\text{sample}}\left(‰\right)=\left(\frac{R_{\text{sample}}}{R_{\text{standard}}}-1\right)\;\times \;1000,$$

where *R*_sample_ is the isotopic ratio of the sample (^2^H/^1^H, ^18^O/^16^O or ^15^N/^14^N) and *R*_standard_ is that of the reference standard, that is, Vienna Standard Mean Ocean Water (δ‰ ^2^H and ^18^O, V‐SMOW) and atmospheric nitrogen (δ‰ ^15^N, N_air_).

##### 2.3.5.1 Determination of Natural Abundance Isotopic Signatures

To determine the natural abundance of ^15^N in foliage, and ^2^H and ^15^N in xylem water, we sampled the study trees on 11 and 12 July, respectively, before tracer application. As reference, samples from three trees of each species and site were averaged (Table S1). Given the highly enriched tracers, isotopic fractionation or background fluctuations were considered to have a negligible influence on the detection of tracers in the tree, making pre‐labelling conditions a suitable baseline.

##### 2.3.5.2 Determination of Tracer Incorporation

When assessing ^2^H enrichment after labelling, we used the LMWL as determined from throughfall measurements to calculate line‐conditioned excess (lc‐excess) (Landwehr and Coplen 2006), for Winnefeld and Unterlüß, respectively. The resulting LMWL were

$\delta^{2}H=5.16\times \delta^{18}O-10.47$ (Winnefeld, *R* ^2^ = 0.80), and

$\delta^{2}H=6.01\times \delta^{18}O-4.60$ (Unterlüß, *R* ^2^ = 0.85).

We further calculated atom‐% excess:

$$\text{atom}-\%\;\text{excess}=\text{atom}-{\%}_{\text{labelled}\;\text{sample}}–\text{atom}-{\%}_{\text{natural}\;\text{abundance}\left(\text{mean}\right),}$$

where we used the ratio of ^15^N to ^2^H atom‐% excess in xylem water samples to assess enrichment of the two tracers relative to each other. The deviation of this ratio from the ratio of the initially applied tracer solution (Ratio_tracer_ = 51.2) served as an indicator for the decoupling of ^15^N and ^2^H translocation. Samples that showed no tracer enrichment were not included in this analysis.

Lastly, we calculated the amount of ^15^N tracer that was incorporated into foliage of each tree canopy at the end of the experiment (14 week after labelling). For foliage mass estimations, we used allometric functions for mixed forests (Forrester et al. 2017).

### 2.4 Models and Statistics

Statistical analyses and plotting was done using R (R Core Team 2025). The effects of site (sandy, loamy), species identity (DF, B) and campaign (4, 5, 6 or 14 weeks) on isotopic signatures were determined using linear mixed models from the package *lme4* (Bates et al. 2015), with a respective subset of data and natural abundance data as reference. Effects that were not significant on their own or in interactions were removed from the model according to ‘backward selection’ procedure (Dorrmann and Kühn 2011).

When assessing the site effect, only DF data was used (since there were no beech trees available on the loamy site). In the model, site was included as fixed effect, with tree ID and campaign as random effects. To address species identity effects, only data from the sandy site (Unterlüß) was used, again with tree ID and campaign as random effects.

The effect of sampling time (factor ‘campaign’) was considered in a set of models independently for each species and site (i.e., DF loamy, DF sandy, B sandy), including tree ID as a random effect. All models were run separately for each of the three response variables (δ^15^N in foliage, δ^15^N in xylem water, or lc‐excess in xylem water).

Finally, we analysed the simultaneous occurrence of ^2^H and ^15^N in xylem water at site and species level with Spearman correlations, using the package *car* (Fox and Weisberg 2019). Graphics were produced with the packages *ggplot2* (Villanueva and Chen 2019), *ggeffects* (Lüdecke 2018), *coefplot* (Lander 2022), *cowplot* (Wilke 2025) and *ggpubr* (Kassambara 2023).

## 3 Results

### 3.1 Meteorological Conditions

**Figure 1:**
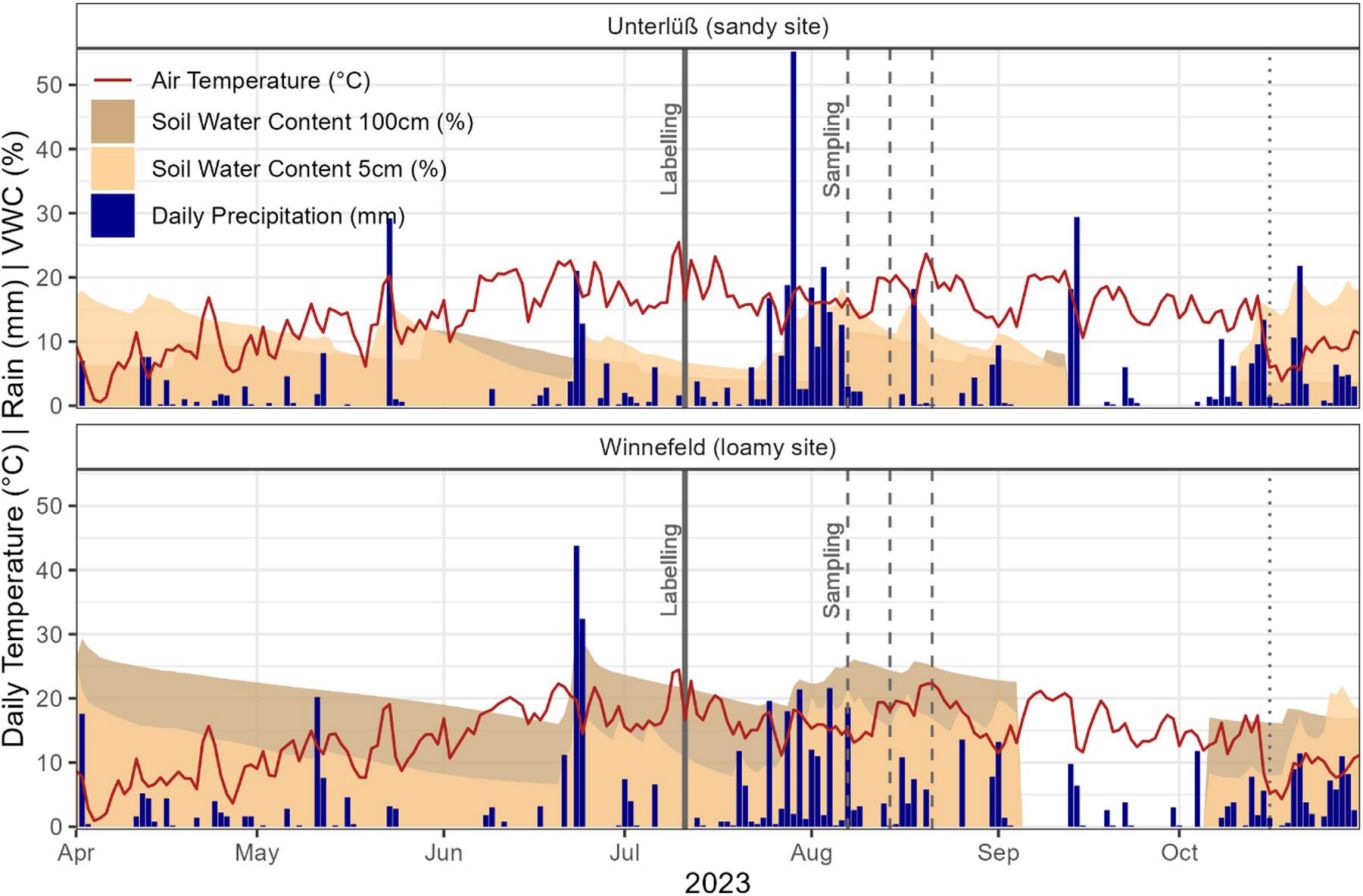
Weather conditions at the two sites during the growing season 2023. Brown shaded area: soil volumetric water content (%) at 5 cm mineral soil depth (light shading) and 100 cm (dark shading) (plot‐level for Douglas fir in Winnefeld, average of the three plots in Unterlüß); blue bars: daily precipitation (mm); red line: daily mean air temperature (°C). There was no soil water data available from 5 September–5 October (Winnefeld) to 13 September–10 October (Unterlüß) due to technical failure of the batteries. The bold solid line marks the labelling date (11 July), which also included natural abundance sampling of xylem water and foliage. Dashed lines represent water and foliage sampling, and the dotted line represents the additional foliage sampling.

The experiment took place in the relatively warm but wet year 2023, with annual precipitation sums and mean annual temperatures notably exceeding long‐term averages on both sites (Unterlüß: 911 mm, 11.5°C [long‐term: 747 mm, 9.0°C]; Winnefeld: 1297 mm, 9.91°C [long‐term: 839 mm, 8.8°C]). The vegetative season (April–October) accounted for 62% and 44% of annual precipitation in Unterlüß and Winnefeld, respectively. There was considerable precipitation on both sites between labelling and sampling (Figure 1), suggesting that any tracer solution that had not been taken up or assimilated within a few days after labelling, likely leached out of the rooting zone.

### 3.2 Tree Water Status

Tree water status as measured by midday xylem water potential remained relatively constant between measurement dates, apart from the first sampling after labelling on the sandy site, which happened after a phase of strong rainfall, and showed less negative Ψ_MD_ for both species. On the loamy site, the second sampling campaign after labelling showed the least negative Ψ_MD_ (Figure S4). On average, we measured Ψ_MD_ = −1.1 ± 0.4 MPa for DF on the loamy site, Ψ_MD_ = −1.9 ± 0.3 MPa for DF on the sandy site, and Ψ_MD_ = −1.5 ± 0.4 MPa for beech on the sandy site (mean ± standard deviation). In accordance with the rather warm and wet weather conditions, we thus expected normal metabolic activity in the trees during the measurement period, for instance, no drought‐induced impairment of tree physiological processes (McDowell et al. 2008).

### 3.3 Soil N Availability and Tree Nutritional Status

Soil available nitrate and nitrate in topsoil solution were highest in the loamy DF stand (Table 2) and lowest in the sandy DF/beech stand. Needle N content of DF was highest in the sandy DF/beech stand. In the mixture, beech had significantly higher foliage N (*p* < 0.05) compared to the pure beech stand, while the needle N content for DF tended to be lower. The needle N:P ratio, an indicator for nutritional imbalance (Mellert and Göttlein 2012), was highest in the loamy DF stand. However, values were still below 16, which is considered the threshold N:P ratio of DF for P deficiency (Coleman et al. 2014). Foliage N:P ratio for beech at the sandy site was close to the upper limit (18.9) still considered balanced for beech (Mellert and Göttlein 2012). The foliage N:P ratio for DF implied a better nutritional status of the trees at the sandy site, particularly in the pure stand, while all DF trees were ranging below the considered limit of 16. Xylem water N content, measured four times in July–August 2023, was highest in sandy DF (22.1 ± 5.4 µg mL^−^^1^), and lower in loamy DF (17.0 ± 4.9 µg mL^−^^1^) and sandy beech (16.1 ± 4.6 µg mL^−^^1^) (Figure S6).

**Table 2:** Bulk soil N availability at 5 cm mineral soil depth estimated with the CaCl_2_ extraction. For soil, four subsamples per plot (2500 m^2^) were averaged, ± indicates standard error of the mean. For foliage the samples of the first campaign were used (7 and 8 August 2023), see Methods. Foliage N % is based on dry biomass mass, foliage N:P ratio is a mass ratio. Soil solution was sampled in July 2021, see Mrak et al. (2024).

Site	Stand type	NH_4_‐N (mg/kg soil)	NO_3_‐N (mg/kg soil)	NO_3_‐N in soil solution at 5 cm (mg/L)	NO_3_‐N in soil solution at 60 cm (mg/L)	Foliage *N* (%)	Foliage N:P
Loamy	DF	0.79 ± 0.16	3.10 ± 2.16	6.76 ± 0.96	6.58 ± 0.52	1.25 ± 0.02	13.91 ± 0.28
Sandy	DF	1.38 ± 0.14	1.26 ± 0.33	4.77 ± 1.41	0.72 ± 0.24	1.16 ± 0.05	9.77 ± 0.46
	DF in DF/B	1.06 ± 0.12	0.16 ± 0.03	0.09 ± 0.02	0.16 ± 0.06	1.11 ± 0.02	11.40 ± 0.05
	B in DF/B					2.25 ± 0.04	18.64 ± 0.13
	B	1.26 ± 0.21	0.36 ± 0.19	0.08 ± 0.03	0.22 ± 0.10	2.00 ± 0.01	17.98 ± 0.31

### 3.4 Transport Velocities of Tracer in the Xylem Sap

The estimated sap travel times to sampling height (20 m in beech and 25 m in DF) using cumulative maximum sap velocity differed among the studied trees, ranging from 23 to 73 days, with a median of 32 days (Figure S5, Table S2). Thus, our sampling 28, 35 and 42 days after labelling occurred around the time when the tracer arrived in almost all tree canopies. The beech tree with an estimated travel time of 73 days was excluded from the analysis, since it was unlikely that the tracer could have appeared during the sampling period. Estimations for two DFs lay close to the last sampling day (44 and 46 days, respectively). Given that measurements of sap velocity tend to overestimate travel time (Seeger and Weiler 2021), and that we found signs of tracer enrichment in these trees, we kept them in the analysis. In case of foliage, which was sampled one last time in mid‐October (14 weeks after labelling), the tracer—if taken up—should have arrived in the canopy of all trees. No sap flow measurements were conducted at the loamy site; however, because the trees were of similar dimensions and we detected tracer in the samples, we assumed that the time window for sampling was transferable.

### 3.5 Water and Nitrate Uptake

**Figure 2:**
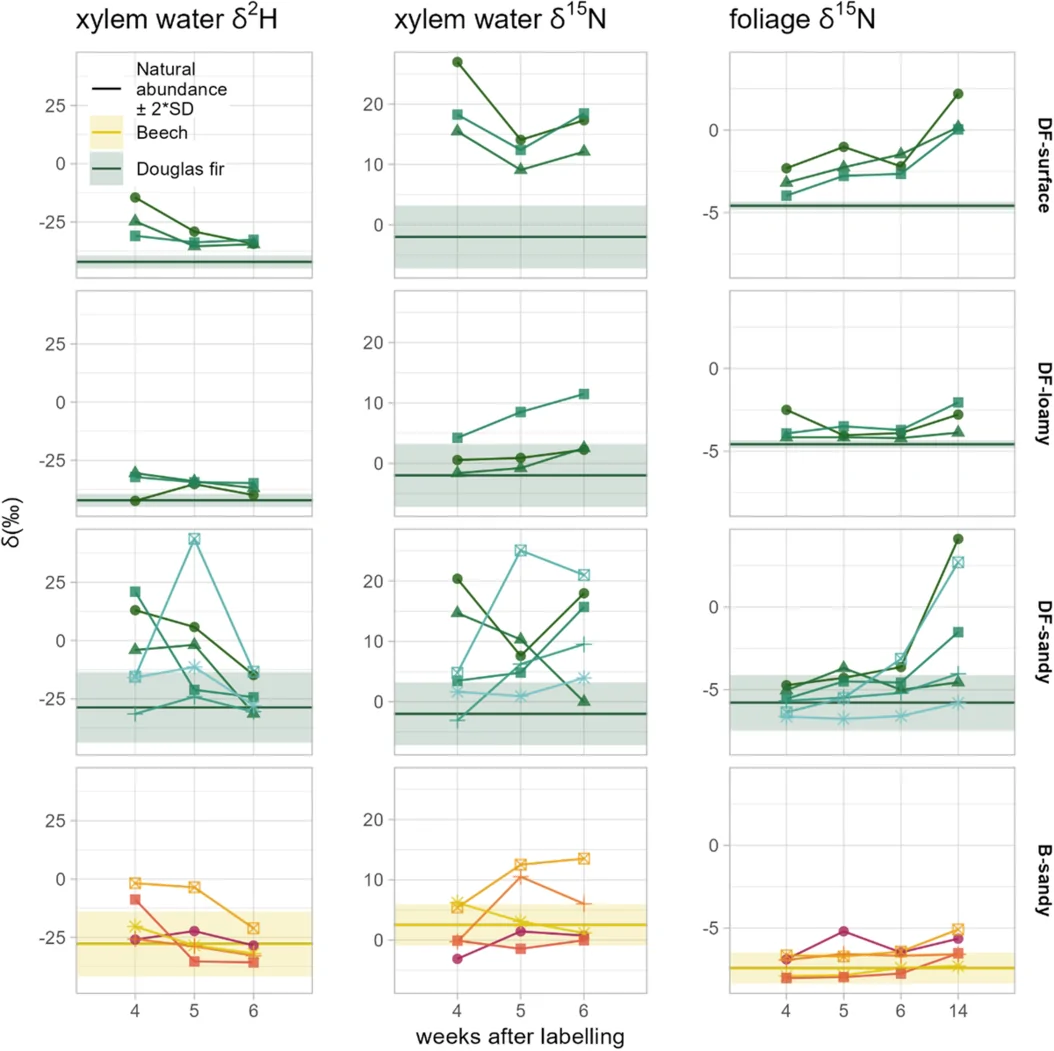
Tracer appearance in xylem water (^2^H and ^15^N, left and middle column) and foliage (^15^N, right column) 4, 5, 6 and 14 weeks after label application for each tree by plot. B = European beech (yellow/orange), DF = Douglas fir (green). For DF, two sites were analysed (sandy, loamy). At the loamy site, one DF plot was labelled at the soil surface (first row), while all other plots were labelled at 60 cm mineral soil depth. Points indicate individual measurements for a particular sampling date; lines connecting points indicate measurements on the same tree. Within a plot (one row), one colour refers to the same individual tree. Horizontal lines are natural abundance means (sampled before labelling) and shading indicates their double standard deviations for the respective species and site.

After applying the tracer solution, we measured δ^2^H (Figure 2, left column) and δ^15^N (Figure 2, central column) in xylem water 4–6 weeks after labelling, and δ^15^N in foliage (Figure 2, right column) 4, 5, 6 and 14 weeks after labelling. As expected, trees subjected to surface labelling generally showed a stronger enrichment than the trees on the neighbouring plot with subsoil labelling.

**Figure 3:**
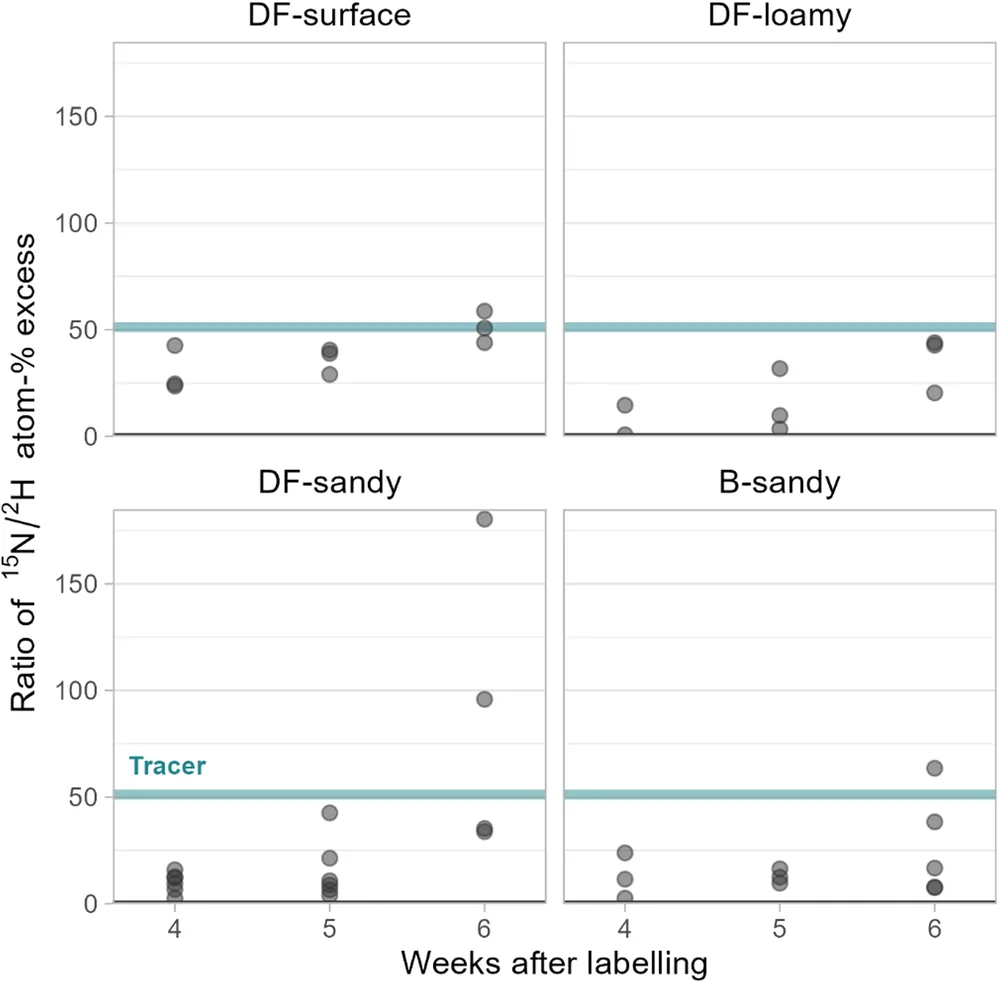
Ratio of ^15^N to ^2^H enrichment (atom‐% excess) in tree xylem water over time (weeks after labelling), separated by tree species and site (with subsoil‐labelling: DF‐sandy = Douglas fir at sandy site, B‐sandy = beech at sandy site, DF‐loamy = Douglas fir at loamy site; with surface‐labelling: DF‐surface = Douglas fir at the loamy site). The thick blue line marks the ratio of the tracer (51.2); lower values indicate relatively higher enrichment of ^2^H (a value of 0 would reflect no ^15^N enrichment, regardless of ^2^H), while higher values indicate relatively higher enrichment of ^15^N. For samples without any tracer enrichment, the ratio cannot be computed, and they are not shown.

Ratios of ^15^N to ^2^H atom‐% excess in xylem water increased over time for both species (Figure 3), indicating a temporal decoupling of the two tracers from labelling to appearance in the canopy. At the first sampling campaign, all trees exhibited relatively higher ^2^H‐enrichment than ^15^N‐enrichment compared to the tracer solution. At the last sampling campaign, 6 weeks after labelling, the xylem water ratio approached or exceeded the tracer ratio, indicating an increasing appearance of the ^15^N tracer in the canopy. For DF, the surface‐labelled trees exhibited less deviation from the tracer, that is, stronger coupling of ^15^N and ^2^H translocation than the subsurface‐labelled trees. Furthermore, the ratio increase was stronger at the sandy than at the loamy site.

**Figure 4:**
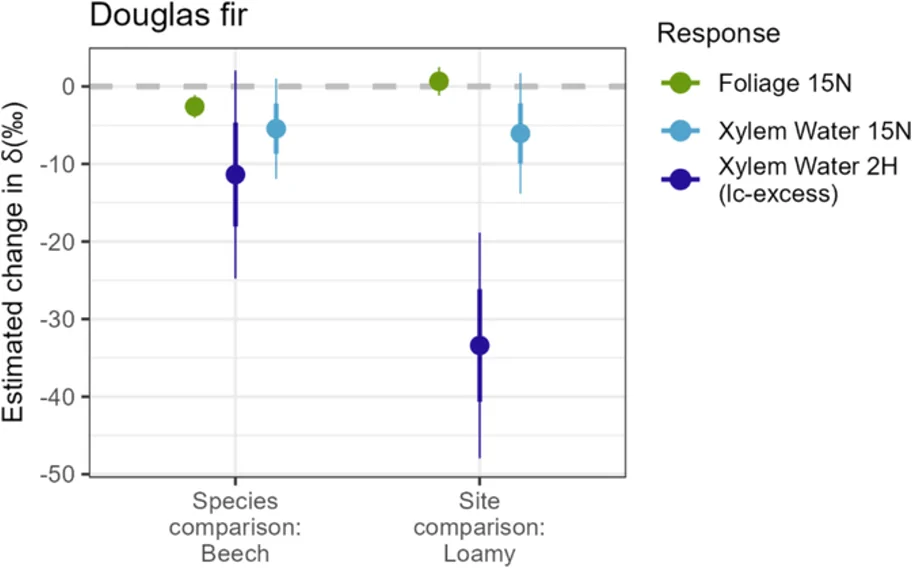
Effects of site and species on tracer signal as estimated by the linear mixed effects models. The reference line (zero effect) represents Douglas fir growing on the sandy site. Based on this scenario, we show the effect of two different fixed effects that were considered in separate models: species (as compared to European beech; shown on the left and including data only from the sandy site) and site (as compared to the loamy site; shown on the right and including only Douglas fir data). A negative change in δ‰ indicates an isotopically more depleted sample. In both models, time and tree ID were considered as random effects. Colours indicate the respective response: green = foliage ^15^N, light blue = xylem water ^15^N, dark blue = xylem water ^2^H. Dots represent the respective model estimates, uncertainties are visualised as thick tails (single standard deviation) and thin tails (double standard deviation).

**Figure 5:**
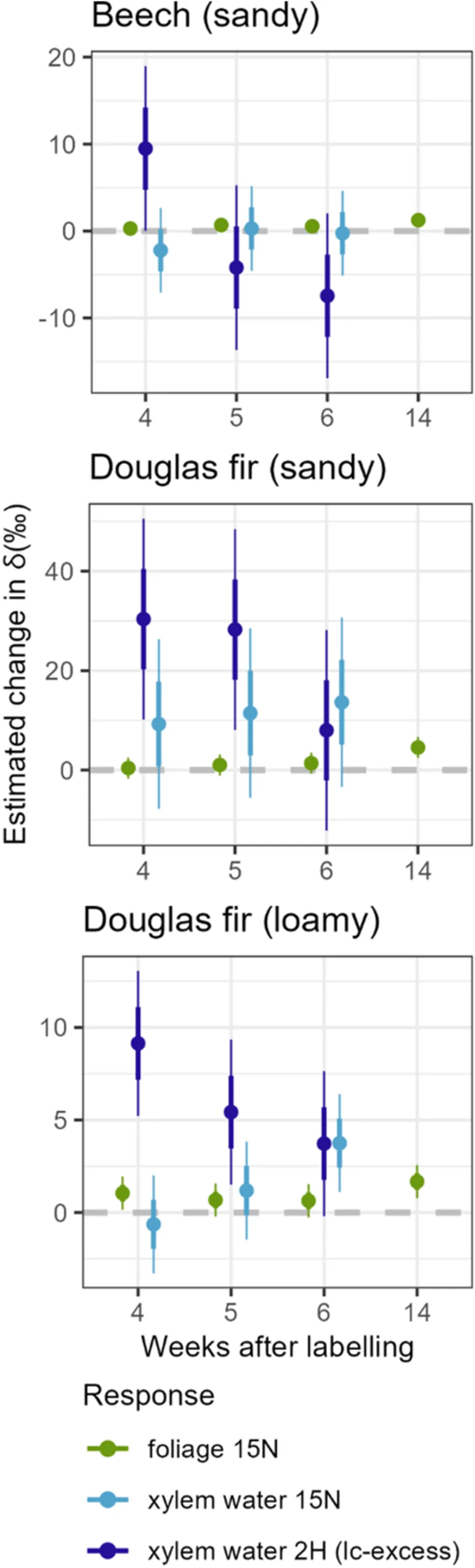
Effects of sample timing on tracer signal, separately for each species (and site). The reference line (zero effect) represents natural abundance before labelling. The estimated change in tracer signal after 4, 5, 6 and 14 weeks (foliage only) as resulting from linear mixed effect models is shown. Colours indicate the respective response: green = foliage ^15^N, light blue = xylem water ^15^N, dark blue = xylem water ^2^H. Dots are respective model estimates, thick tails show single standard deviations, thin tails show double standard deviations.

Measurements made on the subsoil‐labelled plots were analysed using linear mixed models, separately focusing on the effects of site (Figure 4, Tables S3–S5), species identity (Figure 4, Tables S6–S8) and timing (Figure 5, Tables S9–S17) on tracer discovery. All model summaries can be found in Tables S3–S17.

Subsoil uptake of DF was higher at the sandy site than at the loamy site for lc‐excess (*p* < 0.001) (Figure 4, right). Site differences for the ^15^N tracer were not significant (*p* = 0.133 and *p* = 0.481 for xylem water and foliage, respectively). Compared to beech, DF incorporated more ^15^N into foliage (*p* < 0.001); however, the higher presence of ^15^N and ^2^H in xylem water was not significant (*p* = 0.102 and *p* = 0.103 for lc‐excess and ^15^N, respectively) (Figure 4, left).

The timing of discovery was different between tracers (^2^H and ^15^N in xylem water) and compartments (^15^N in xylem water and foliage) (Figure 5). ^15^N in foliage increased over time, with a significant enrichment in all stand types (DF loamy, DF sandy, B sandy) after 14 weeks, highlighting its sink function. The ^2^H tracer in xylem water was detected in all stand types at the first sampling 4 weeks after labelling (*p* = 0.003, *p* = 0.008, *p* = 0.062 for DF loamy, DF sandy, B sandy, respectively), and persisted in the following week for both DF stands (*p* = 0.033, *p* = 0.012 for DF loamy and DF sandy, respectively). After 6 weeks, no significant enrichment was detected any more. The ^15^N tracer in DF xylem water did not follow the same temporal pattern as ^2^H; concentrations increased throughout the sampling period (Figures 2, 5). For beech, the ^15^N tracer was not detectable in xylem water. Further, we did not observe strong correlations between lc‐excess and δ^15^N in the same xylem water samples taken after labelling (Figure S11).

The patterns of ^15^N in xylem water among species and sites mirrored the total N content in xylem water (Figures S7,S8); DF on the sandy site showed higher N concentrations than both beech on the sandy site, and DF on the loamy site. Based on sap flow (available only on the sandy site) and xylem water N concentrations, we estimated a total N import into the above‐ground biomass of 51.3 ± 23.2 g per beech tree, and 228.9 ± 116.2 g per DF tree from the day of labelling until the last foliage sampling (14 weeks afterwards), equivalent to 5.5 ± 2.4 and 2.4 ± 1.4 g N kg^−1^ foliage biomass for beech and DF, respectively.

### 3.6 ^15^N Tracer Recovery in Foliage

The amount of nitrogen tracer that was recovered in foliage per g dry mass and per tree canopy after 14 weeks was larger for DF than for beech (Figures S9,S10). For DF, we recovered on average more tracer per g biomass and per canopy on the sandy site (7.58%; 12.80 mg per canopy) than on the loamy site (3.18%; 5.37 mg per canopy). The recovery per g dry DF biomass was similar on the mixed and pure sandy plots (0.19 and 0.22 ug/g dry foliage, respectively), but by per canopy estimation, the recovery was slightly higher at the mixed plot (11.44%; 19.32 mg and 7.58%; 12.80 mg per canopy). For beech, we recovered more tracer in foliage on the mixed plot (0.67%; 1.12 mg per canopy) than on the pure plot (0.37%; 0.63 mg per canopy).

## 4 Discussion

To our knowledge, the present study is the first to demonstrate the decoupling of water and nitrate uptake in mature forest trees under field conditions, with nitrogen reaching the canopy later than water. Furthermore, we highlight the impact of site conditions, which are often disregarded when investigating species identity effects on resource uptake (Schulz et al. 2011; Wang and Macko 2011). We identified water and nitrogen uptake patterns of DF, a non‐native species increasingly gaining importance in European silviculture, at two contrasting sites. At one site, we drew the comparison to European beech, the naturally dominating tree species of temperate European forests. We show that it is crucial to consider site conditions when evaluating tree species functional traits driving water and nutrient uptake, whereas tree species mixture did not affect the outcome in the investigated setting.

### 4.1 De‐Coupling of Water and Nitrogen Uptake

The appearance of simultaneously applied tracers in xylem sap followed different temporal patterns, with ^15^N appearing much later than ^2^H (Figures 3, 5), supporting our first hypothesis. This delay was not species‐specific, as it occurred in both DF and beech (Figure 3). Nor was it dependent on labelling depth, as the same pattern was observed for DF labelled both at the surface and deeper soil layers. However, the surface labelling generally resulted in a higher ratio of ^15^N to ^2^H, implying a tighter coupling between water and nitrate uptake in topsoil.

In root tips, mycorrhizal nutrient retention may delay or even inhibit the translocation to host tissue (Rivera Pérez et al. 2022; Audisio et al. 2024). In the root tissue, nitrate is rapidly reduced to ammonium and assimilated into amino acids (Finlay et al. 1989; Yoneyama and Suzuki 2019), further delaying the translocation of N from fine roots to the stem and foliage compared to water. Further, ectomycorrhizal fungi (ECM) may modulate tree water and N uptake differently. While the direct effect of ECM on the root hydraulic conductivity of trees and thus their impact on water uptake is not fully understood (Coleman et al. 1990; Lehto and Zwiazek 2011), the effect of ECM on nitrate acquisition can be large and delay its transit time (Warren et al. 2008; Hawkins and Kranabetter 2017). The observed variations in tree water and nitrogen uptake might also be related to different ECM diversity and abundance between the sites (Likulunga et al. 2021), which have distinct N uptake capacities and preferences (Khokon et al. 2023) influencing the retention, transport and metabolism of nitrogenous compounds (Pena and Tibbett 2024).

These findings suggest that the asynchronicity in nitrogen (N) and water uptake is likely an infrastructural feature of the soil‐plant system. It encompasses multiple factors facilitating or inhibiting the uptake of N, including plant physiological and root functional traits (Hernando et al. 2025; Göransson et al. 2008), seasonality (Lucash et al. 2005), microbial competition and mycorrhizal interaction (Hasselquist et al. 2016; Audisio et al. 2024), as well as soil properties, specifically moisture, texture and resource availability (Dail et al. 2001; Kreuzwieser and Gessler 2010; Kulmatiski et al. 2017).

The generally long transit time—even of the deuterium tracer—from subsoil to tree canopy observed in our study (Figure S5, Table S2) and previous studies (e.g., Magh et al. 2020; Werner et al. 2021; Hackmann et al. 2025) highlights a key aspect of tree water and solute transport. Even under active transpiration, the time taken for newly absorbed molecules to reach the upper canopy may range from weeks up to months depending on tree species and size, individual sap velocities and environmental conditions. This time may be extended through exchange with internal water (Ziemińska et al. 2020; Hernando et al. 2025) or nitrogen reserves (Herschbach et al. 2012; Babst and Coleman 2018), as well as radial and azimuthal variation in sap flow (Čermák et al. 2004; Cohen et al. 2008), which explains why the sap flow measurements could approximate, but not exactly predict the arrival of the tracer. As a result, water and nitrogen tracers applied to the soil may take several weeks to be detectable in the upper canopy, which is crucial for budgeting, and designing experimental studies on mature trees.

### 4.2 Greater Subsoil Water and Nitrate Uptake for Douglas Fir at Sandy Sites

DF took up more water from the subsoil on the sandy than on the loamy site (Figure 4), supporting our second hypothesis and demonstrating that uptake patterns of trees are site‐dependent (Miller and Hawkins 2003; Martins et al. 2009). The observed higher subsoil N uptake by DF on the sandy than the loamy site was statistically insignificant. Still, higher fine root biomass and root area index (Lwila et al. 2021), as well as a lower nitrate leaching risk (Mrak et al. 2024) were observed by previous studies on the sandy compared to the loamy site, suggesting that in the sandy, resource‐poor environment, resources were used more effectively across soil depths. In contrast, nitrate availability appears to exceed demands at the loamy site (Mrak et al. 2024), and our findings suggest that tree water and N demand were largely met by uptake from the topsoil, where N concentrations and root uptake capacity are higher (Dannenmann et al. 2016; Castro‐Rodríguez et al. 2017; Lwila et al. 2021).

### 4.3 Greater N and Water Uptake From Subsoil by Douglas Fir Than by European Beech

The uptake of both tracers from the subsoil was higher in DF than in beech at the sandy site (Figures 3, 4), leading us to reject hypothesis 3. At this site, beech is deeper‐rooting than DF and also develops higher fine root biomass and root area index at the labelled depth in the subsoil, but particularly in shallower soil layers (Lozanova et al. 2019; Lwila et al. 2021; Pietig et al. 2026). Thus, it appears that beech may have covered most of its N and water requirements from the topsoil instead of relying on deeper roots, while DF was using resources from both pools. This may also be related to the higher total tree‐level water and nitrogen uptake of DF, with its greater size and larger leaf area (Paligi et al. 2025). Given the higher water uptake and sap N concentrations of DF compared to beech (Figure S6), tree‐level N uptake of DF was approximately four times higher (Figure S8), suggesting that N uptake across a larger depth profile was necessary and plausible.

Different patterns of internal N cycling in deciduous beech and evergreen DF might be a reason for the observed differences in ^15^N uptake and incorporation into foliage (Gebauer et al. 2000). Possibly, beech was less responsive than DF to the added tracer because it is better able to adapt to a changing N availability on the whole tree level (Geβler et al. 2004) by reducing its dependency on current mineral N supply by storage and remobilisation processes (Millard and Grelet 2010). Furthermore, contrasting seasonal N dynamics in the foliage might have played a role. During our last sampling date at the end of the season, it is likely that the process of N re‐translocation from leaves to storage tissues in the stem and roots was already under way in beech (Figure S12 and Rennenberg et al. 2010), which often starts in the sun leaves as early as mid‐summer (Gessler et al. 1998).

Species preferences in N uptake form likely have influenced our results (Ashton et al. 2010; Dannenmann et al. 2016). We applied N as nitrate, and compared the two species at the sandy site, where ammonium naturally dominates (Table 2). While both beech and DF can absorb N in different forms, the uptake differs depending on N form preferences, availability and ratios of the N forms, season and tree species interactions (Gessler et al. 1998; Simon et al. 2021; Rivera Pérez et al. 2022; Uscola et al. 2017; Mrak et al. accepted). For instance, the low nitrate uptake of beech at the sandy site may reflect a preference for organic N compounds over nitrate under low N availability (Dannenmann et al. 2009; Stoelken et al. 2010; Simon et al. 2021). Observations of one N form therefore provide an incomplete picture of N uptake patterns. Yet, nitrate's high solubility and weak soil binding make it suitable for coupling analyses of N and water uptake (Hachiya and Sakakibara 2017).

## 5 Conclusions

Synchronous labelling of water and nitrate demonstrates that adult DF and beech trees take up both resources at different rates and that the water is transported up the xylem more rapidly than the N. This may be attributable to delayed N uptake, which could occur due to differences between mass flow (water) and diffusion (N) in the soil, the interaction of biota with N during its passage through the soil, different modes of transport through root membranes (aquaporins vs. high‐ and low‐affinity nitrate transporters), and the physiological fate of nitrate in the plant. It is also plausible that a tree possesses roots that are primarily responsible for nutrient uptake (e.g., the fine roots in the organic layer) and others that are mainly active in water uptake (as the deep roots), pointing at a functionally dimorphic root system. The observed large influence of soil type (sandy vs. loamy) on nitrate and water uptake patterns of DF demonstrates that tree functional traits derived from the species’ morphological properties (such as specific root length) can be misleading if site differences are neglected. Future research should aim for characterising the functional role of deep roots and for quantitative modelling of deep resource use in forests, integrating ecophysiological processes across scales. Since roots are multifunctional organs, a straightforward way to investigate their functioning is by measuring uptake rates of different resources synchronously; a blueprint for this is provided by the present study.

## Funding

This study was supported by the Deutsche Forschungsgemeinschaft.

## Supporting information

MrakHackmann Decoupled Supplementary.

## Data Availability

The data that support the findings of this study are openly available in GRO.data at https://data.goettingen-research-online.de/, https://doi.org/10.25625/IW0XME.
