# Supplementary material for: De‐Coupled Water and Nitrogen Translocation From Subsoil to Canopy of Temperate Forest Trees
Source: Plant Cell Environ. 2025 Dec 15;49(10):7676–91. doi: 10.1111/pce.70327 (PMC13551642; doi:10.1111/pce.70327)
Supplement: Supplementary file 1 [file PCE-49-7676-s001.docx]

**De-coupled water and nitrogen translocation from subsoil to canopy of temperate forest trees**

Mrak, Hackmann et al.

# Supplementary material

Table of contents

**Fine root biomass	3**

Figure S1	3

**Radial sap flux density profile	4**

Figure S2	4

**Study design	5**

Figure S3	5

**Midday twig water potentials	6**

Figure S4	6

**Estimating tracer travel time from sap flow measurements	7**

Figure S5	7

Table S2	8

**N in xylem water	9**

Figure S6	9

Figure S7	9

**Total N import into aboveground biomass	10**

Figure S8	10

**Foliage mass estimation and N tracer recovery	11**

Methods S1	11

Figure S9	12

Figure S10	12

**Correlation of tracer signals	13**

Figure S11	13

**N concentration in litter over time	14**

Figure S12	14

**Natural abundance	15**

Table S1

**Model outputs	16**

Site Effects

Table S3	16

Table S4	16

Table S5	17

Species identity effects

Table S6	17

Table S7	18

Table S8	18

Timing effects

Table S9	19

Table S10	19

Table S11	20

Table S12	20

Table S13	21

Table S14	21

Table S15	22

Table S16	22

Table S17	23

**References	24**

## Fine root biomass

**Figure S1**. Fine root live biomass distribution with soil depth of each species on the investigated plots (Pure Douglas fir in Winnefeld (loamy site, top left) and Unterlüß (sandy site, top right), pure beech in Unterlüß (sandy site, bottom right), and mixed beech-Douglas fir in Unterlüß (sandy site, bottom left)). Redrawn from (Lwila et al., 2021) with permission.

## Radial sap flux density profiles

**Figure S2**. Sap flux density proportion (%) in sap wood by depth (cm) for European beech (B, left) and Douglas fir (DF, right) on pure plots (top) and on the mixed plot (bottom). Dashed lines represent 10% of the maximum sap flux density proportion estimated for each tree (adapted from Paligi et al. 2025).

## Study Design

**Figure S3**. Schematic representation of the experimental design. Dots represent tree stems; dashed circles represent circles on which the labelling tubes were installed (8 per circle, 2 m radius around the stem). D = Douglas fir, B = European beech.

## Midday twig water potentials

**Figure S4**. Midday twig water potentials measured at the same sampling dates as the stable isotopes. Panels separate the species, with beech in the upper panel and Douglas fir in the lower panel. Symbols indicate the plot type (DF = Douglas fir pure stand, DF/B = Douglas fir/European beech mixed stand, B = European beech pure stand, DF control = surface labelling at the loamy site). Colors indicate the site (brown = loamy site I(Winnefeld), yellow = sandy site (Unterlüß). Each data point is labelled with the tree ID number, which corresponds to the numbers used in Figure S5 and Table S2.

## Estimating tracer travel time from sap flow measurements

**Figure S5**. Sap velocity (dotted line, m day^-1^) and cumulative sap travel height (solid line, m) from the date of labelling (11 July 2023) to the last day of foliage sampling (16 October 2023) for each sample tree on the Unterlüß site. The three dashed lines indicate sampling dates for xylem water and foliage. Colored horizontal lines indicate sampling height (beech (yellow): 20 m, Douglas fir (green): 25 m) and can be referred to cumulative sap travel height. After the day when the solid line crossed sampling height, the tracer was assumed to be detectable in the samples. Sap velocity was calculated from the maximum sap flux density within the tree-specific radial sap wood profile. Sap flow measurements of one Douglas fir (D092) were not considered due to sensor malfunction.

**Table S2**: Estimated minimum tracer travel time to sampling height (Beech: 20m, Douglas fir: 25m) from sap flow measurements for each sample tree on the Unterlüß site. Xylem water sampling was conducted 4, 5, and 6 weeks (28, 35, and 42 days) after labelling. Trees with travel times greater than until the last sampling campaign are highlighted in bold. Estimations of two Douglas fir trees were very close to the last xylem sampling date on 21 August (highlighted in bold italic), and we did recover tracer in these trees, so we kept them in the analysis. One beech tree (B056) however, showed overall low sap velocities and no tracer recovery, so we excluded it from the analysis, assuming that the tracer could not have arrived in the canopy during the sampling period.

| Tree | Estimated tracer arrival at sampling height | Species | Stand type | Estimated tracer travel time after labelling (days) |
| --- | --- | --- | --- | --- |
| B066 | 2023-08-03 | Beech | B | 23 |
| B143 | 2023-08-16 | Beech | B | 36 |
| B002 | 2023-08-20 | Beech | B | 40 |
| B129 | 2023-08-03 | Beech | DF/B | 23 |
| B054 | 2023-08-05 | Beech | DF/B | 25 |
| **B056** | **2023-09-22** | **Beech** | **DF/B** | **73** |
| D051 | 2023-08-11 | Douglas fir | DF/B | 31 |
| ***D053*** | ***2023-08-24*** | ***Douglas fir*** | ***DF/B*** | ***44*** |
| ***D125*** | ***2023-08-26*** | ***Douglas fir*** | ***DF/B*** | ***46*** |
| D062 | 2023-08-05 | Douglas fir | DF | 25 |
| D037 | 2023-08-12 | Douglas fir | DF | 32 |
| D092 | NA | Douglas fir | DF | NA |

## N in xylem water

**Figure S6**. Total N content per mL xylem water. Green is species DF, yellow is species B. Plots: DF = Douglas fir pure stand, DF/B = Douglas fir/European beech mixed stand, B = European beech pure stand. Control = surface labelling at the loamy site. Dots are observations from all sampling campaigns after labelling.

**Figure S7**. Isotopic signature of nitrogen in xylem water. Green is species DF, yellow is species B. Plots: DF = Douglas fir pure stand, DF/B = Douglas fir/European beech mixed stand, B = European beech pure stand. Control = surface labelling at the loamy site. Dots are observations from all sampling campaigns after labelling.

## Total N import into aboveground biomass

N (g)

**Figure S8**. Cumulative tree-level N uptake (import into aboveground biomass) since labelling for the trees on the sandy site (Unterlüß), calculated based on sap flow (Liters) and N concentration in sap. Colors indicate different trees.

## Foliage mass estimation and N tracer recovery

**Methods S1**. We used data from a field-inventory realized in 2018, determining the diameter at breast height (DBH) and species of each tree and using them in allometric equations. We used a different method for each species; for Douglas fir, we used allometric equations from (Ishii et al., 2002), linking branch needle dry weight to branch length in mature Douglas fir. For European beech, foliage mass estimations were done using allometric estimations for foliage mass from DBH (Forrester et al., 2017).

The ^15^N tracer recovery in tree biomass was estimated by scaling isotopic

concentration with allometric equations described above, as follows: first, the isotopic excess above natural abundance was multiplied by the N content in biomass and by the biomass factor (from the allometric equations) to produce the amount of tracer per canopy. Then, the tracer recovered in each canopy was expressed as a percentage of the total tracer applied around each canopy (168.88 mg).

**Figure S9**. Amount of tracer recovered per g dry foliage biomass after 14 weeks. Calculated as foliage N concentration * excess atom fraction. Green is species DF, yellow is species B. Plots: DF = Douglas fir pure stand, DF/B = Douglas fir/European beech mixed stand, B = European beech pure stand. Small dots are observations of trees, large dots represent means.

**Figure S10**. Amount of tracer recovered per one tree canopy after 14 weeks. Calculated as foliage N concentration * excess atom fraction * canopy foliage mass. Green is species DF, yellow is species B. Plots: DF = Douglas fir pure stand, DF/B = Douglas fir/European beech mixed stand, B = European beech pure stand. Small dots are observations of trees, large dots represent means.

### Correlation of tracer signals in tree xylem water

**Figure S11**: Correlations of lc-excess and δ15N tracer in tree xylem water after labelling, coloured by species. Points indicate individual tree measurements. Spearman’s rho (ρ) and p-value (p) are reported for each species and site (DF loamy, DF sandy, B sandy).

**N concentration in litter over time**

**Figure S12**: Nitrogen concentration in newly fallen litter by stand type (both sites together) over the course of the vegetative season in 2023. Litter collection is described in Methods in the main text. DF = Douglas fir pure stand, DF/B = Douglas fir/European beech mixed stand, B = European beech pure stand. Litter was sampled monthly in litter traps (60 ×40×20 cm), with 2 cm and a mesh separating them from the forest floor, with 4 traps on pure plots, and six traps on mixed plots, positioned under a closed canopy on a diagonal transect of the plot. The samples were dried at 60°C for 72 hours, milled and analysed on a C:N analyzer (vario MICRO cube CN analyzer, Elementar Analysensysteme, GmbH, Langenselbold Germany).

## Natural abundance

| (a) Xylem water ^2^H (δ‰) | | |  |  |
| --- | --- | --- | --- | --- |
| Site | Species | mean | | sd |
| Loamy | DF | -42.099 | | 1.385 |
| Sandy | DF | -28.623 | | 7.567 |
|  | B | -27.839 | | 6.953 |
| (b) Xylem water ^15^N (δ‰) | | |  |  |
| Site | Species | mean | | sd |
| Loamy | DF | -2.009 | | 2.600 |
| Sandy | DF | -2.009 | | 2.600 |
|  | B | 2.528 | | 1.719 |
| (c) Foliage ^15^N (δ‰) | | |  |  |
| Site | Species | mean | | sd |
| Loamy | DF | -4.577 | | 0.119 |
| Sandy | DF | -5.785 | | 0.831 |
|  | B | -7.420 | | 0.468 |

**Table S1**. **Natural abundance** in xylem water (a, b) and in foliage (c) for ^2^H (a) and ^15^N (b, c), given in δ‰ V-SMOW. Mean = average of three trees of a species (b) or three trees of a species per site (a, c), sd = standard deviation. DF = Douglas fir, B = European beech.

## Model outputs

1. Site effects

Each tracer was analyzed separately in a linear mixed effects model, with Species as predictor, and campaign (weeks after labelling) and tree ID as random effects. Only Douglas fir data was included. Below, the model outputs are listed as tables.

**Table S3**. Lc-excess.

|  | **Effects on xylem water 2H (lc-excess)** | | |
| --- | --- | --- | --- |
| *Predictors* | *Estimates* | *CI* | *p* |
| (Intercept) | 18.96 | 6.51 – 31.40 | **0.005** |
| Quintet [3] | -33.4 | -48.49 – -18.32 | **<0.001** |
| **Random Effects** | | | |
| σ^2^ | 231.23 | | |
| τ_00_ _Tree_ | 28.73 | | |
| τ_00_ _campaign_ | 55.13 | | |
| ICC | 0.27 | | |
| N _campaign_ | 3 | | |
| N _Tree_ | 9 | | |
| Observations | 27 | | |
| Marginal R^2^ / Conditional R^2^ | 0.450 / 0.596 | | |

**Table S4**. ^15^N in xylem water.

|  | **Effects on xylem water 15N** | | |
| --- | --- | --- | --- |
| *Predictors* | *Estimates* | *CI* | *p* |
| (Intercept) | 9.16 | 4.38 – 13.94 | **0.001** |
| Quintet [3] | -6.06 | -14.12 – 2.00 | 0.133 |
| **Random Effects** | | | |
| σ^2^ | 35.64 | | |
| τ_00_ _Tree_ | 18.33 | | |
| τ_00_ _campaign_ | 0.85 | | |
| ICC | 0.35 | | |
| N _campaign_ | 3 | | |
| N _Tree_ | 9 | | |
| Observations | 27 | | |
| Marginal R^2^ / Conditional R^2^ | 0.134 / 0.437 | | |

**Table S5**. ^15^N in foliage

|  | **Effects on foliage 15N** | | |
| --- | --- | --- | --- |
| *Predictors* | *Estimates* | *CI* | *p* |
| (Intercept) | -4.23 | -5.90 – -2.55 | **<0.001** |
| Quintet [3] | 0.66 | -1.23 – 2.55 | 0.481 |
| **Random Effects** | | | |
| σ^2^ | 2.86 | | |
| τ_00_ _Tree_ | 1 | | |
| τ_00_ _campaign_ | 1.55 | | |
| ICC | 0.47 | | |
| N _campaign_ | 4 | | |
| N _Tree_ | 9 | | |
| Observations | 36 | | |
| Marginal R^2^ / Conditional R^2^ | 0.018 / 0.481 | | |

2. Species identity effects

Each tracer was analyzed separately in a linear mixed effects model, with Site as predictor, and campaign (weeks after labelling) and tree ID as random effects. Only data from Unterlüß (the sandy site) was included. Below, the model outputs are listed as tables.

**Table S6**. Lc-excess.

|  | **Effects on xylem water 2H (lc-excess)** | | |
| --- | --- | --- | --- |
| *Predictors* | *Estimates* | *CI* | *p* |
| (Intercept) | 18.96 | 4.45 – 33.46 | **0.012** |
| Species [Beech] | -11.35 | -25.09 – 2.38 | 0.102 |
| **Random Effects** | | | |
| σ^2^ | 204.18 | | |
| τ_00_ _Tree_ | 54.58 | | |
| τ_00_ _campaign_ | 89.12 | | |
| ICC | 0.41 | | |
| N _campaign_ | 3 | | |
| N _Tree_ | 11 | | |
| Observations | 33 | | |
| Marginal R^2^ / Conditional R^2^ | 0.087 / 0.464 | | |

**Table S7**. ^15^N in xylem water.

|  | **Effects on xylem water 15N** | | |
| --- | --- | --- | --- |
| *Predictors* | *Estimates* | *CI* | *p* |
| (Intercept) | 9.16 | 4.61 – 13.72 | **<0.001** |
| Species [Beech] | -5.45 | -12.07 – 1.18 | 0.103 |
| **Random Effects** | | | |
| σ^2^ | 33.18 | | |
| τ_00_ _Tree_ | 17.47 | | |
| τ_00_ _campaign_ | 0.57 | | |
| ICC | 0.35 | | |
| N _campaign_ | 3 | | |
| N _Tree_ | 11 | | |
| Observations | 33 | | |
| Marginal R^2^ / Conditional R^2^ | 0.129 / 0.436 | | |

**Table S8**. ^15^N in foliage.

|  | **Effects on foliage 15N** | | |
| --- | --- | --- | --- |
| *Predictors* | *Estimates* | *CI* | *p* |
| (Intercept) | -4.23 | -5.74 – -2.71 | **<0.001** |
| Species [Beech] | -2.59 | -4.11 – -1.08 | **0.001** |
| **Random Effects** | | | |
| σ^2^ | 2.42 | | |
| τ_00_ _Tree_ | 0.92 | | |
| τ_00_ _campaign_ | 1.23 | | |
| ICC | 0.47 | | |
| N _campaign_ | 4 | | |
| N _Tree_ | 11 | | |
| Observations | 44 | | |
| Marginal R^2^ / Conditional R^2^ | 0.272 / 0.614 | | |

3. Timing after labelling effects

Each tracer was analyzed separately for each species and site in a linear mixed effects model, with campaign (weeks after labelling) as predictor, and tree ID as random effect. Below, the model outputs are listed as tables.

3.1 Beech (sandy site)

**Table S9**. Lc-excess, beech (sandy site).

|  | **Effects on xylem water 2H (lc-excess)** | | |
| --- | --- | --- | --- |
| *Predictors* | *Estimates* | *CI* | *p* |
| (Intercept) | 7.82 | -2.41 – 18.05 | 0.125 |
| campaign [4_Weeks] | 9.48 | -0.56 – 19.53 | 0.062 |
| campaign [5_Weeks] | -4.2 | -14.24 – 5.84 | 0.388 |
| campaign [6_Weeks] | -7.44 | -17.48 – 2.60 | 0.136 |
| **Random Effects** | | | |
| σ^2^ | 51.22 | | |
| τ_00_ _Tree_ | 56.43 | | |
| ICC | 0.52 | | |
| N _Tree_ | 6 | | |
| Observations | 22 | | |
| Marginal R^2^ / Conditional R^2^ | 0.300 / 0.667 | | |

**Table S10**. ^15^N in xylem water, beech (sandy site).

|  | **Effects on xylem water 15N** | | |
| --- | --- | --- | --- |
| *Predictors* | *Estimates* | *CI* | *p* |
| (Intercept) | 3.74 | -1.65 – 9.14 | 0.16 |
| campaign [4_Weeks] | -2.21 | -7.41 – 2.98 | 0.378 |
| campaign [5_Weeks] | 0.29 | -4.90 – 5.49 | 0.906 |
| campaign [6_Weeks] | -0.25 | -5.44 – 4.94 | 0.919 |
| **Random Effects** | | | |
| σ^2^ | 10.91 | | |
| τ_00_ _Tree_ | 13.73 | | |
| ICC | 0.56 | | |
| N _Tree_ | 6 | | |
| Observations | 21 | | |
| Marginal R^2^ / Conditional R^2^ | 0.043 / 0.576 | | |

**Table S11**. ^15^N in foliage, beech (sandy site).

|  | **Effects on foliage 15N** | | |
| --- | --- | --- | --- |
| *Predictors* | *Estimates* | *CI* | *p* |
| (Intercept) | -7.48 | -8.16 – -6.79 | **<0.001** |
| campaign [4_Weeks] | 0.3 | -0.30 – 0.89 | 0.314 |
| campaign [5_Weeks] | 0.69 | 0.10 – 1.29 | **0.025** |
| campaign [6_Weeks] | 0.56 | -0.04 – 1.15 | 0.066 |
| campaign [14_Weeks] | 1.25 | 0.66 – 1.85 | **<0.001** |
| **Random Effects** | | | |
| σ^2^ | 0.19 | | |
| τ_00_ _Tree_ | 0.35 | | |
| ICC | 0.65 | | |
| N _Tree_ | 6 | | |
| Observations | 28 | | |
| Marginal R^2^ / Conditional R^2^ | 0.242 / 0.734 | | |

3.2 Douglas fir (sandy site)

**Table S12**. Lc-excess, Douglas fir (sandy site).

|  | **Effects on xylem water 2H (lc-excess)** | | |
| --- | --- | --- | --- |
| *Predictors* | *Estimates* | *CI* | *p* |
| (Intercept) | -3.26 | -19.43 – 12.92 | 0.676 |
| campaign [4_Weeks] | 30.37 | 9.08 – 51.67 | **0.008** |
| campaign [5_Weeks] | 28.27 | 6.97 – 49.57 | **0.012** |
| campaign [6_Weeks] | 8 | -13.30 – 29.30 | 0.439 |
| **Random Effects** | | | |
| σ^2^ | 276.57 | | |
| τ_00_ _Tree_ | 17.87 | | |
| ICC | 0.06 | | |
| N _Tree_ | 6 | | |
| Observations | 23 | | |
| Marginal R^2^ / Conditional R^2^ | 0.367 / 0.406 | | |

**Table S13**. ^15^N in xylem water, Douglas fir (sandy site).

|  | **Effects on xylem water 15N** | | |
| --- | --- | --- | --- |
| *Predictors* | *Estimates* | *CI* | *p* |
| (Intercept) | -2.29 | -19.84 – 15.26 | 0.782 |
| campaign [4_Weeks] | 9.26 | -9.15 – 27.68 | 0.297 |
| campaign [5_Weeks] | 11.45 | -6.96 – 29.87 | 0.202 |
| campaign [6_Weeks] | 13.64 | -4.77 – 32.06 | 0.133 |
| **Random Effects** | | | |
| σ^2^ | 56.1 | | |
| τ_00_ _Tree_ | 16.09 | | |
| ICC | 0.22 | | |
| N _Tree_ | 6 | | |
| Observations | 19 | | |
| Marginal R^2^ / Conditional R^2^ | 0.123 / 0.318 | | |

**Table S14**. ^15^N in foliage, Douglas fir (sandy site).

|  | **Effects on foliage 15N** | | |
| --- | --- | --- | --- |
| *Predictors* | *Estimates* | *CI* | *p* |
| (Intercept) | -6.07 | -7.97 – -4.17 | **<0.001** |
| campaign [4_Weeks] | 0.4 | -1.80 – 2.60 | 0.709 |
| campaign [5_Weeks] | 1.03 | -1.17 – 3.23 | 0.343 |
| campaign [6_Weeks] | 1.38 | -0.82 – 3.58 | 0.208 |
| campaign [14_Weeks] | 4.55 | 2.35 – 6.75 | **<0.001** |
| **Random Effects** | | | |
| σ^2^ | 3.03 | | |
| τ_00_ _Tree_ | 1.31 | | |
| ICC | 0.3 | | |
| N _Tree_ | 6 | | |
| Observations | 29 | | |
| Marginal R^2^ / Conditional R^2^ | 0.384 / 0.570 | | |

3.3 Douglas fir (loamy site)

**Table S15**. Lc-excess, Douglas fir (loamy site).

|  | **Effects on xylem water 2H (lc-excess)** | | |
| --- | --- | --- | --- |
| *Predictors* | *Estimates* | *CI* | *p* |
| (Intercept) | -20.54 | -24.93 – -16.16 | **<0.001** |
| campaign [4_Weeks] | 9.14 | 4.34 – 13.94 | **0.003** |
| campaign [5_Weeks] | 5.43 | 0.63 – 10.23 | **0.033** |
| campaign [6_Weeks] | 3.72 | -1.07 – 8.52 | 0.106 |
| **Random Effects** | | | |
| σ^2^ | 5.77 | | |
| τ_00_ _Tree_ | 3.86 | | |
| ICC | 0.4 | | |
| N _Tree_ | 3 | | |
| Observations | 12 | | |
| Marginal R^2^ / Conditional R^2^ | 0.550 / 0.731 | | |

**Table S16**. ^15^N in xylem water, Douglas fir (loamy site).

|  | **Effects on xylem water 15N** | | |
| --- | --- | --- | --- |
| *Predictors* | *Estimates* | *CI* | *p* |
| (Intercept) | 1.67 | -5.23 – 8.56 | 0.561 |
| campaign [4_Weeks] | -0.63 | -4.04 – 2.77 | 0.652 |
| campaign [5_Weeks] | 1.19 | -2.21 – 4.59 | 0.41 |
| campaign [6_Weeks] | 3.75 | 0.35 – 7.15 | **0.037** |
| **Random Effects** | | | |
| σ^2^ | 1.97 | | |
| τ_00_ _Tree_ | 18.3 | | |
| ICC | 0.9 | | |
| N _Tree_ | 3 | | |
| Observations | 11 | | |
| Marginal R^2^ / Conditional R^2^ | 0.138 / 0.916 | | |

**Table S17**. ^15^N in foliage, Douglas fir (loamy site).

|  | **Effects on foliage 15N** | | |
| --- | --- | --- | --- |
| *Predictors* | *Estimates* | *CI* | *p* |
| (Intercept) | -4.58 | -5.39 – -3.77 | **<0.001** |
| campaign [4_Weeks] | 1.05 | 0.01 – 2.09 | **0.048** |
| campaign [5_Weeks] | 0.68 | -0.36 – 1.71 | 0.17 |
| campaign [6_Weeks] | 0.63 | -0.40 – 1.67 | 0.196 |
| campaign [14_Weeks] | 1.67 | 0.64 – 2.71 | **0.006** |
| **Random Effects** | | | |
| σ^2^ | 0.3 | | |
| τ_00_ _Tree_ | 0.07 | | |
| ICC | 0.18 | | |
| N _Tree_ | 3 | | |
| Observations | 15 | | |
| Marginal R^2^ / Conditional R^2^ | 0.465 / 0.563 | | |

References

Forrester, D.I., Benneter, A., Bouriaud, O., Bauhus, J., 2017. Diversity and competition influence tree allometric relationships – developing functions for mixed‐species forests. Journal of Ecology 105, 761–774. https://doi.org/10.1111/1365-2745.12704.

Ishii, H., Ford, E.D., Boscolo, M.E., Manriquez, A.C., Wilson, M.E., Hinckley, T.M., 2002. Variation in specific needle area of old-growth Douglas-fir in relation to needle age, within-crown position and epicormic shoot production. Tree physiology 22, 31–40. https://doi.org/10.1093/treephys/22.1.31.

Lwila, A.S., Mund, M., Ammer, C., Glatthorn, J., 2021. Site conditions more than species identity drive fine root biomass, morphology and spatial distribution in temperate pure and mixed forests. Forest Ecology and Management 499, 119581. https://doi.org/10.1016/j.foreco.2021.119581.
